# Effects of LED Light on *Acacia Melanoxylon* Bud Proliferation *in Vitro* and Root Growth *ex Vitro*

**DOI:** 10.1515/biol-2019-0039

**Published:** 2019-07-22

**Authors:** Shubin Li, Lili Zhou, Sipan Wu, Li Liu, Meng Huang, Sizu Lin, Guochang Ding

**Affiliations:** 1 College of Arts College of Landscape Architecture, Fujian Agriculture and Forestry University, Fuzhou, 350002, P. R. China; 2 Forestry College, Fujian Agriculture and Forestry University, Fuzhou, 350002, P. R. China; 3 Chinese Fir Engineering Technology Research Center of the State Forestry and Grassland Administration, Fuzhou, 350002, P. R. China; 4 Institute of Oceanography, Minjiang University, Fuzhou, 350108, P. R. China; 5 Forest Park Engineering Research Center of State Forestry and Grassland Administration, Fuzhou 350002, P.R. China

**Keywords:** Acacia melanoxylon, Light-emitting diode (LED) light, Multiple bud proliferation, Plantlet growth, Root growth

## Abstract

This study examines the effects of light emitting diodes (LEDs) on tissue culture proliferation of *Acacia melanoxylon* plantlets among five different clones (FM_1_, FM_2_, FM_4_, FM_5_, and FM_10_). Shoot bud apex cuttings were transplanted onto Murashige and Skoog basal medium containing 0.1 mg L^-1^ 6-benzyladenine and 0.5 mg L^-1^ naphthalene acetic acid and cultured *in vitro* for 40 days. Root growth was studied under different light intensities and photoperiods *ex vitro*. The bud proliferation coefficient was greatest under a light intensity of 45 μmol m^-2^ s^-1^ photosynthetic photon flux and photoperiod of 16 h light, but decreased as the light intensity increased. However, the greatest light intensity was beneficial for the growth of robust plantlets. Plantlets exposed to red and blue LED combinations grew tall and green, with a small number of roots. Plantlets also grew taller and some roots expanded under the longer photoperiod. Increased light intensity had positive effects on root number and rooting rate, and prolonged light greatly increased root number. Therefore, lower light intensity and a short photoperiod were beneficial for bud proliferation, while red/blue LED combinations, increased light intensity, and longer light illumination were beneficial for plantlet growth and root growth of *Acacia melanoxylon*.

## 1 Introduction

Tissue culture plays a major role in the propagation of plants as varied as ornamental foliage crops, medicinal plants, and woody trees with desirable characteristics [1]. Several external and internal factors regulate *in vitro* plant growth and development. Light is the most important of these factors because the illumination system used can affect photosynthesis and the photomorphogenic response [2, 3]. The light environment includes the photon flux density, light quality and photoperiod. Currently, light-emitting diode (LED) technology is widely used as a flexible artificial lighting source with several unique advantages compared to conventional lighting systems like fluorescent lamps, high-pressure sodium lamps, metal halide lamps, and incandescent lamps. LED lighting systems exhibit wavelength specificity, durability, small sizes, long operating lifetimes, and relatively cool emitting surfaces. Because the photon output is linearly related to the electrical input, the spectral composition can be controlled [3, 4]. Several plant species grow well under LEDs, including seedlings of lettuce, pepper, cucumber, wheat, and spinach; plantlets of potato; and *in vitro* cultures of *Rehmannia glutinosa* [5, 6, 7, 8]. Therefore, LED lights are ideal instruments for use in lighting designs for *in vitro* plant production.

*Acacia melanoxylon* is an evergreen tree species belonging to the family Mimosaceae and the genus *Acacia* Mill. It is fast-growing, is tolerant to both drought and barren soil, and has notable nitrogen fixation abilities. It has excellent wood material and is used for superior furniture. Due to its high economic and ecological value, it has been planted in many areas in China ever since it was introduced from Australia in the early 1990s [9]. Because of some serious difficulties with seed breeding, there has been increased attention paid to clonal afforestation. Clonal afforestation can not only keep the parent’s excellent characteristics, but also form a stable forest. Plantlets in tissue culture have high somaclonal variations, are not restricted by external conditions, can produce plantlets several times in one year, and can cover small areas with high propagation coefficients. Therefore, tissue culture propagation is currently a main method of asexual propagation of this species. There have been many reports relating to the establishment of an efficient tissue culture system for rapid propagation of *Acacia melanoxylon* [10, 11]. For example, Hu et al. [12] used aseptic leaves, petioles, and stems of geminated axillaries of *Acacia melanoxylon* to induce calluses and established high-frequency regeneration systems. He found that the optimal material for callus formation was the stem and the optimal medium for induction was Murashige and Skoog basal medium + 1.5 mg L^-1^ 6-benzyladenine + 0.2 mg L^-1^ naphthalene acetic acid + 3% sucrose. Dou et al. [13] reported the genetic diversity of 174 isolates of symbiotic bacteria associated with *Acacia melanoxylon* from different sites in China. However, there has been little research on the effects of the light environment on tissue propagation of *Acacia melanoxylon* [14, 15]. Light intensity, quality, and photoperiod may control the growth and development of tissue organ cultures by triggering physiological reactions.

The purpose of this research was to study the effects of light environment on bud proliferation of *in vitro* tissue cultures of *Acacia melanoxylon*. We hypothesized that (1) LED light intensity, quality, and photoperiod would have effects on bud proliferation and plantlet growth, and (2) LED light intensity and photoperiod would have effects on root growth of *Acacia melanoxylon*. We attempted to determine the optimal light environment and provide some theoretical and technical support for providing this type of lighting to tissue cultures of *Acacia melanoxylon*.

## 2 Materials and Methods

### 2.1 Plants

The research materials were five clones of *Acacia melanoxylon* designated FM_1_, FM_2_, FM_4_, FM_5_, and FM_10_. They were provided by the Chinese Fir Engineering Research Center of the State Forestry Administration, Fujian Agriculture and Forestry University, Fuzhou, Fujian Province.

### 2.2 Experimental design

The experiment was divided into two parts. First, we designed three *in vitro* experiments to test the effects of LED light intensity, quality and photoperiod on bud proliferation and plantlet growth. Second, we designed two *ex vitro* experiments to test the effects of LED light intensity and photoperiod on root growth.

#### 2.2.1 Effects of LED light environment on bud growth and plantlet growth in vitro

We selected strong and healthy buds of similar size from the each of the five clones of *Acacia melanoxylon*. Four tissue clumps were transplanted onto Murashige and Skoog basal medium with 0.1 mg L^-1^ 6-benzyladenine and 0.5 mg L^-1^ naphthalene acetic acid and cultured under different light treatments *in vitro* for 40 days at ~25°C. Six buds formed one tissue clump, and there was 4 tissue clumps in each replicate. There were 10 replicates for each treatment.

The three experiments *in vitro* were as follows: (1) The effects of LED light intensity on bud proliferation and plantlet growth. Plants were cultured under white LED light with a wavelength of 400–700 nm and intensity of 45 μmol m^-2^ s^-1^, 90 μmol m^-2^ s^-1^, or 135 μmol m^-2^ s^-1^ respectively. (2) The effects of LED light quality on bud proliferation and plantlet growth. Because plants need to grow under photosynthetic active radiation between 400 nm and 700 nm, and because photosynthetic efficiency is highest at wavelengths of 450 nm (blue) and 650 nm (red), we designed experimental LED light combinations using red and blue wavelengths. The ratio of red and blue LEDs were 1: 1, 1: 4, and 4: 1, all with an intensity of 135 μmol m^-2^ s^-1^. There was also a control with light provided by a fluorescent lamp. (3) The effects of LED photoperiod on bud proliferation and plantlet growth. We used two photoperiods, 16 h light and 8 h dark or 12 h light and 12 h dark, under 135 μmol m^-2^ s^-1^ white LED light. For each treatment of LED light intensity, light quality, and light photoperiod, the tissues were cultivated under controlled light condition in sterile tissue culture room.

After 40 days, we calculated the bud proliferation coefficient (*K*) and observe plantlet growth for each clone under the different LED intensities, qualities, and photoperiods. The formula was *K* = the number of buds after 40 days/the number of buds at the initiation of the experiment.

#### 2.2.2 Effects of LED light environment on root growth *ex vitro*

The *ex vitro* experiments were based on a substrate of peat, vermiculite, and perlite at a mass ratio of 5: 4: 1. After the substrates were thoroughly mixed, they were poured into 0.1% potassium permanganate solution, mixed, and transferred to pots. The substrates were then washed with water after 3 d, separated into cultivation pots and compressed, then watered as needed. After samples were air-dried and finely ground, pH measurements were made, and each sample was adjusted to pH 5.8. To study light intensity and light photoperiod influences on root growth *ex vitro*, for each light treatment, 30 healthy plantlets without roots (average height ca. 3 cm) from FM_1_, FM_2_, FM_4_, FM_5_, and FM_10_ clones *in vitro* tissue cultures were transferred to the substrate described above. The base roots of samples were not treated using rooting accelerator treatments. All they were then placed in controlled light environment in sterile tissue culture room.

The two experiments *ex vitro* were as follows: (1) The effects of LED light intensity on root growth. We used three light intensities, 45 μmol m^-2^ s^-1^, 90 μmol m^-2^·s^-1^, and 135 μmol m^-2^ s^-1^, with white LED light of wavelength 400–700 nm. The photoperiod was 16 h light and 8 h dark. (2) The effects of LED light photoperiod on root growth. We used two photoperiods, 12 h light and 12 h dark or 16 h light and 8 h dark, with white LED light intensity of 135 μmol·m^-2^·s^-1^ and wavelength of 400–700 nm.

After 30 days, we calculated average rooting rate, average root length, and average number of roots (root number) per plant. The average root length was calculated as the total root length divided by the total number of plants with root formation. Rooting rate was calculated as the number of plants with root formation divided by the total number of plants.

### 2.3 Statistical analysis

A one-way ANOVA was used to compare the differences between treatments and to evaluate the effects of LED light intensity, quality, and photoperiod on bud proliferation coefficient, average root length, and rooting rate. Mean comparisons were performed with the least significant difference (LSD) test at *p* = 0.05. All statistical analyses were performed with the SPSS statistical package (SPSS 12.0, SPSS Inc., IL., USA).

## 3 Results

### 3.1 The effects of LED light intensity on bud proliferation and plantlet growth in vitro

The effects of LED light intensity on bud proliferation of *Acacia melanoxylon* are shown in Table 1. The LED light intensity had significant effects on FM_2_, FM_4_, and FM_5_ (*p*< 0.01), as well as on FM_1_ (*p*< 0.05). LED light intensity had no significant effects on FM_10_ (*p* = 0.8001). For FM_1_ and FM_2_, the bud proliferation coefficient decreased with increasing light intensity, and it was significantly highest with light intensity of 45 μmol m^-2^ s^-1^. For FM_4_, the coefficient varied significantly among the three light intensities, and the maximum bud proliferation coefficient appear at the light intensity of 45 μmol m^-2^ s^-1^. For FM_5_, the coefficient was significantly higher with the light intensity of 90 μmol m^-2^ s^-1^ than with the other two intensities. For FM_10_, there was no significant difference among the three light intensities. Therefore, bud proliferation coefficient was highest under the light intensity of 45 μmol m^-2^ s^-1^ for all the clones except for FM_5_.

**Table 1:** LSD multiple comparison of different light intensities on bud proliferation coefficient of five clones.

Treatment	Light intensity (μmol m^-2^ s^-1^)	Average bud proliferation coefficient				
		FM_1_	FM_2_	FM_4_	FM_5_	FM_10_
1	45	6.64a	7.15a	7.09a	6.34b	6.44a
2	90	5.12ab	4.75b	4.00b	8.08a	6.13a
3	135	3.83b	4.40b	5.10c	6.09b	6.33a

Note: Different lowercase letters in the same column indicate significant differences between different LED light intensities for the same clone (*p*< 0.05).

**Figure 1:**
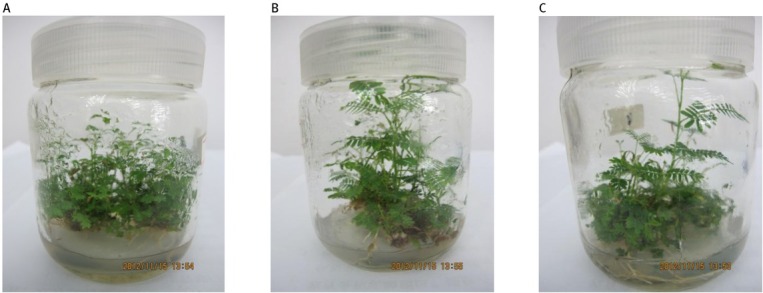
Plantlet growth of FM_2_ clone under different LED intensities. (A) 45 μmol·m^-2^·s^-1^, (B) 90 μmol·m^-2^·s^-1^ and (C) 135 μmol·m^-2^·s^-1^.

We found that plantlet growth was well-supported under all tested LED light intensities (Figure 1). All plantlets were strong and grew to an average length of 2–3 cm and all plantlets grew roots, though the plantlets under 135 μmol m^-2^ s^-1^ had more root number and rooting rate. Therefore, the *Acacia melanoxylon* plantlets grew stronger and higher with the increased light intensities, but with lower bud proliferation coefficient.

### 3.2 The effects of LED light quality on bud proliferation and plantlet growth *in vitro*

The effects of red and blue LED light combinations on bud proliferation of *Acacia melanoxylon* are shown in Table 2. The bud proliferation coefficient decreased from the red/ blue light ratio combination of 1:1 to 4:1 and 1:4, and the average proliferation coefficient was all lower than the control treatment. However, there were only significantly lower with the red/blue ratio of 1:4 compared to other two red/blue combinations on the bud proliferation coefficient. Under the treatment of a red/blue ratio of 4: 1, the plantlets grew higher and with a small amount of root growth; however under the treatment of a red/blue ratio of 1: 4, the plantlets grew with a produced green leaves but without root growth. Therefore, compared to the fluorescent lamp control treatment, the plantlets under the red/blue LEDs grew well, but bud proliferation was poor (Table 2).

**Table 2:** LSD multiple comparison of the effects of light quality on bud proliferation coefficient of five clones.

Treatments	Light quality	Average bud proliferation coefficient				
		FM_1_*	FM_2_*	FM_4_*	FM_5_*	FM_10_*
control	Fluorescent lamp	4.22a	4.66a	5.01a	5.13a	5.46a
1	red/blue LED ratio (1:1)	3.71ab	3.93b	4.02b	4.14b	4.43b
2	red/blue LED ratio (1:4)	2.81c	3.02c	3.11b	3.32c	3.51c
3	red/blue LED ratio (4:1)	3.22ab	3.36b	3.77b	3.64b	4.10b

Note: Mean±SD. Different lowercase letters in the same column indicate significant differences between different light qualities for the same clone at *p < 0.05.

### 3.3 The effects of LED light photoperiod on bud proliferation and plantlet growth *in vitro*

For FM_2_ and FM_4_, the effects of LED light photoperiod on bud proliferation were highly significant (*p* < 0.01); for FM_5_, the effects were significant (*p* < 0.05); and for FM_1_ and FM_10_, the effects were not significant (*p* = 0.05) (Table 3). The bud propagation coefficient was significantly higher in the treatment with the photoperiod of 12 h light and 12 h dark (*p* < 0.05) for FM_2_, FM_4_, and FM_5_. We found that the photoperiod of 12 h light was beneficial for the bud proliferation; however, the photoperiod of 16 h light may promote a small number of roots growth and promote greater plantlet height (Figure 3). Therefore, prolonged the photoperiod of light illumination may decreased bud proliferation, but improve the height growth and root growth of plantlets.

**Table 3:** LSD multiple comparison of the effect of photoperiod on bud proliferation coefficient of five clones.

Clones	Photoperiod (light/dark)	Average bud proliferation coefficient
FM_1_	12h/12h	6.64a
	16h/8h	5.13a
FM_2_*	12h/12h	7.15a
	16h/8h	4.20b
FM_4_*	12h/12h	7.09a
	16h/8h	3.28b
FM_5_*	12h/12h	6.34a
	16h/8h	3.90b
FM_10_	12h/12h	6.44a
	16h/8h	6.19a

Note: Mean±SD. Different lowercase letters indicate significant differences between different photoperiods for the same clone at *p < 0.05.

**Figure 2:**
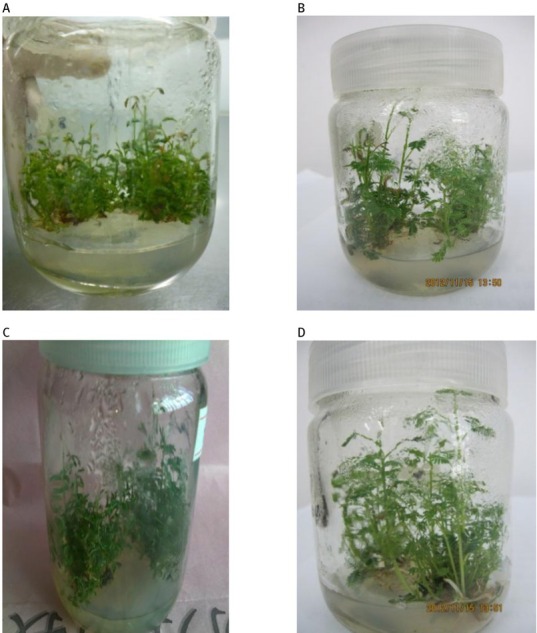
Plantlet growth of FM_2_ clone under fluorescent lamp and different red/blue light ratio combinations. (A) fluorescent lamp, (B) ratio 1: 1, (C) ratio 1: 4 and (D) ratio 4: 1.

**Figure 3:**
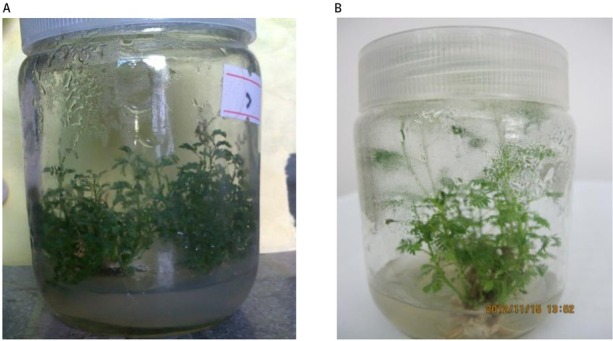
Plantlet growth of FM_2_ clone under different LED light photoperiods. (A) 12 h light and 12 h dark and (B) 16 h light and 8 h dark.

### 3.4 The effects of light intensity on root growth *ex vitro*

We found that the average root number and rooting rate significantly increased with increasing light intensity (Table 4) (*p*< 0.05), while the average root length decreased, though there were no significant differences for FM_1_ and FM_10_ (*p*= 0.05). The average root number was 5–13 among the five clones. The average root length was highest in FM_4_ at 2.57 cm, while the average rooting rate was highest in FM_2_ at 90%.

**Table 4:** Effects of different light intensity on root growth *ex vitro*.

Clones	Light intensity (μmol m^-2^ s^-1^)	Average root numbers (numbers/plant)	Average root length (cm/one root)	Average rooting rate (%)
FM_1_	45	6.34c	1.52a	73.33c
	90	7.10b	1.44a	80.00b
	135	8.04a	1.21a	91.11a
FM_2_	45	6.51c	1.79a	81.11c
	90	7.09b	1.46ab	91.11b
	135	9.00a	1.28b	97.78a
FM_4_	45	5.86c	2.89a	81.11c
	90	6.48b	2.66a	88.89b
	135	8.52a	2.17b	96.67a
FM_5_	45	6.00c	l.77a	82.22b
	90	8.25b	1.56ab	83.33b
	135	11.48a	1.26b	90.00a
FM_10_	45	5.29c	1.43a	80.00b
	90	7.44b	1.31a	83.33b
	135	13.34a	1.13a	96.67a

Note: Mean±SD. Different lowercase letters indicate significant differences between different photoperiods for the same clone (*p* < 0.05).

### 3.5 The effects of photoperiod on root growth *ex vitro*

We found that a prolonged photoperiod did not have significant effects on average root length or average rooting rate (Table 5) (*p*= 0.05) but did have a significant effect on average root numbers (*p*< 0.05). Prolonging the light photoperiod significantly increased average root numbers, but to some extent decreased average root length. The effects of light photoperiod on root numbers were more strongly observed in FM_5_ and FM_10_ compared to the other clones. In particular, the highest average root number was 13 in FM_10_ with 16 h light and 8 dark.

**Table 5:** Effects of photoperiod on root growth *ex vitro*.

Clones	Photoperiod (light/dark)	Average root number (roots/ plant)	Average root length (cm/one root)	Average rooting rate (%)
FM_1_	12h/12h	7.37b	1.39a	91.11a
	16h/8h	8.04a	1.21a	91.11a
FM_2_	12h/12h	7.88b	1.41a	97.78a
	16h/8h	9.00a	1.28a	97.78a
FM_4_	12h/12h	6.97b	2.33a	97.78a
	16h/8h	8.52a	2.17a	96.67a
FM_5_	12h/12h	4.52b	1.85a	90.00a
	16h/8h	11.48a	1.77a	90.00a
FM_10_	12h/12h	6.38b	1.24a	96.67a
	16h/8h	13.34a	1.13a	96.67a

Note: Mean±SD. Different lowercase letters indicate significant differences between different photoperiods for the same clone (*p* < 0.05).

## 4 Discussion

Our results partly verified our hypothesis that LED light intensity, quality, and photoperiod would have great effects on bud proliferation and plantlet growth of *Acacia melanoxylon*. We found that LED light intensity had significant effects on bud proliferation of clones FM_1_, FM_2_, FM_4_, and FM_5_ (*p*< 0.05) and the maximum proliferation coefficient was under the light intensity of 45 μmol m^-2^ s^-1^. The proliferation coefficient decreased with increasing light intensity, but the plantlets grew well with green leaves and strong stems under increased light intensity. Our results were consistent with other reports. Lin [16] reported that high light intensity had negative effects on bud differentiation, perhaps due to light-induced stress. However, compared to our result that the best light intensity for *Acacia melanoxylon* growth was 135 μmol m^-2^ s^-1^, Li et al. [17] found that stem diameter, fresh weight, average diameter, root volume, and root tips of *Dendrobium officinale* Kimura et Migo *in vitro* were significantly higher under light intensity of 75 μmol m^-2^ s^-1^ than under 50 μmol m^-2^ s^-1^ or 100 μmol m^-2^ s^-1^. The difference between the responses of these two species may be that *Acacia melanoxylon* is a light-demanding species and *Dendrobium officinale* is a shade-demanding species.

In our study, we found that bud proliferation was poor under red/blue LED light compared to fluorescent lamp treatments, but plantlets under the red/blue ratio 4:1 grew taller and produced some roots (Table 2, Figure 2). Moon et al. [18] reported rooting is promoted by red LEDs and inhibited by blue LEDs during the study of LEDs effects on Tsuru-rindo cultured *in vitro*. Red LEDs had a positive effect on plantlet height and root growth according to Kong et al. [19] who studied the effects of light quality on the growth of *in vitro* cultured *Doritaenopsis* plants. Our results were in accord with Qiu et al. [20], who reported that LED light combinations were beneficial for axillary bud height and had significant effects on maximum leaf width and fresh leaf mass, while fluorescent light was suitable for *Tectonagrandis* cultures and rapid differentiation of axillary buds. Zhou et al. [21] similarly found that red LED light was advantageous to the growth of aerial parts of plants, and that blue light had effects on both dwarfed and strong tissue culture seedlings of *Anoectochilus roxburghii*. It has been reported that a mixture of blue and red LEDs enhanced plant growth as well as fresh and dry weights compared to monochromatic LEDs [22, 23]. Chen et al. [24] indicated that leaf length, blade width, and root number reached their maximums with a red/blue light ratio of 8:2, which is consistent to our findings (the plantlets under a red/blue ratio of 4: 1 grew higher and with a small amount of root growth). Similar results with Red:Blue (1:1) were also obtained from *in vitro* plants of *Lillium* [25], banana [26] and strawberry [7]. We found the red/blue combination of LED lights (1: 1) was beneficial for the bud proliferation coefficient but increasing the red ratio (R: B = 4:1) could enhance plantlets height and root growth. Nhut and Nam [27] reported that the blue LEDs under 10% (30% in some cases) may promote plant height growth. Further studies need to focus on identifying an optimal red/blue combination of LEDs for both bud proliferation and plantlet growth for *Acacia melanoxylon*.

We found that extending the photoperiod could decrease the proliferation coefficient of buds of *Acacia melanoxylon* (Table 3). However, we also found that longer illumination was beneficial for plantlet height and root growth. Our results were in agreement with Zhang et al. [28], who reported that the average height, average root collar diameter, and average numbers of lateral buds and lateral shoots of *Abies concolor* were all greatest with 22 h light at 50 μmol m^-2^ s^-1^. Some studies found that plants grown at more Northern latitudes, high elevation, and in a continental climate were very sensitive to photoperiod. Tinus [29] reported that extending the photoperiod in the greenhouse was beneficial for the height growth of southern Rocky Mountain Engelmann Spruce and Douglas-fir.

In our study, the average root number and average rooting rate increased with increasing light intensity, and the optimal light intensity was 135 μmol·m^-2^·s^-1^ (Table 4). In root growth experiments, plantlets changed from photoheterotrophic growth to photoautotrophic growth. The light intensity was neither excessively high nor low; strong light intensity may destroy the chlorophyll [30]. We found that increased photoperiod could increase average root numbers but decrease root length (Table 5). These results were similar to Li et al. [31], who indicated that 10 h light per day was enough for sweet pepper plantlets, but prolonging the light was beneficial for root growth. However, Fan et al. [32] found that if the photoperiod was longer or shorter than 16 h, the shoot-rooting of *Paulownia* plants was inhibited. Kurilcik et al. [33] studied the effects of illumination spectrum on the chrysanthemum plantlets grown using LED lights, and found that blue LEDs enhanced plantlet extension and roots formation. Therefore, we will continue to study the effects of light quality (blue, red and far-red) on *Acacia melanoxylon* propagation in the future.

## 5 Conclusions

In our study, we found that the optimal light intensity for bud proliferation of the clones FM_1_, FM_2_, FM_4_, FM_5_, and FM_10_ of Chinese fir *Acacia melanoxylon* was 45 μmol m^-2^ s^-1^. Higher light intensity was not beneficial for bud proliferation, but with increasing light intensity, plantlet growth was improved. The effects of red/blue combinations of LEDs on bud proliferation were not significant, but plantlet and root growth was better under red/blue LEDs ratio 4:1 than under a fluorescent lamp. The extended photoperiod was not beneficial for bud proliferation, but good for the plantlet growth of *Acacia melanoxylon in vitro*. Increased light intensity greatly increased root number and the rooting rate *ex vitro*, while a prolonged photoperiod greatly increased root number. In short, increases in light intensity and photoperiod were beneficial for plantlet growth and root numbers, but much less so for bud proliferation; a red/blue LED ratio of 4:1 was advantageous to plantlet and root growth.
